# Dense Space-Division Multiplexing Exploiting Multi-Ring Perfect Vortex

**DOI:** 10.3390/s23239533

**Published:** 2023-11-30

**Authors:** Xing Liu, Duo Deng, Zhenjun Yang, Yan Li

**Affiliations:** 1 College of Physics, Hebei Key Laboratory of Photophysics Research and Application, Hebei Normal University, Shijiazhuang 050024, China; liuxing@stu.hebtu.edu.cn (X.L.); zjyang@hebtu.edu.cn (Z.Y.); 2 Department of Optoelectronics Science, Harbin Institute of Technology, Weihai 264209, China; liy@hit.edu.cn

**Keywords:** optical vortex, orbital angular momentum, free space optical communication, atmospheric turbulence

## Abstract

Vortex beams carrying orbital angular momentum (OAM) have gained much interest in optical communications because they can be used to expand the number of multiplexing channels and greatly improve the transmission capacity. However, the number of states used for OAM-based communication is generally limited by the imperfect OAM generation, transmission, and demultiplexing methods. In this work, we proposed a dense space-division multiplexing (DSDM) scheme to further increase the transmission capacity and transmission capacity density of free space optical communications with a small range of OAM modes exploiting a multi-ring perfect vortex (MRPV). The proposed MRPV is generated using a pixel checkerboard complex amplitude modulation method that simultaneously encodes amplitude and phase information in a phase-only hologram. The four rings of the MRPV are mutually independent channels that transmit OAM beams under the condition of occupying only one spatial position, and the OAM mode transmitted in these spatial channels can be efficiently demodulated using a multilayer annular aperture. The effect of atmospheric turbulence on the MRPV was also analyzed, and the results showed that the four channels of the MRPV can be effectively separated under weak turbulence conditions. Under the condition of limited available space and OAM states, the proposed DSDM strategy exploiting MRPV might inspire wide optical communication applications exploiting the space dimension of light beams.

## 1. Introduction

Since Allen and coworkers discovered that a vortex beam carries orbital angular momentum (OAM) of *lћ* per photon in 1992 [1], the generation, detection, and application technologies of OAM have been widely developed [2,3,4,5,6,7,8]. With the continuous efforts of researchers, OAM has been applied in many fields such as optical tweezers [9], microscopy [10], quantum optics [11], remote sensing [12], chiral spectroscopy [13,14], and optical communications [15]. Here, *l* is the topological charge (TC) of the vortex beam, and *ћ* is the reduced Planck constant. Such beams have an azimuthal phase structure exp(*ilϕ*), where *ϕ* is the azimuthal angle. For the conventional vortex with a TC that is an integer, OAM beams with different *l* are intrinsically orthogonal when they propagate coaxially. Therefore, these spatially orthogonal modes are desired as additional dimensions in optical communications [16]. Space-based communication technology, which employs spatially orthogonal modes or spatial positions to increase the multiplexing channels, is called space-division multiplexing (SDM) [17]. SDM can be used in both optical fibers and free space to greatly increase the transmission capacity of optical communication systems. For fiber optical communications, high-capacity OAM-based SDM exploiting multi-ring fibers has been demonstrated [18], and the transmission capacity is mainly limited by the performance of fibers [19]. When the SDM technique is used in free-space optical communications, the optical communication links are affected by atmospheric turbulence [20] and the divergence of OAM beams [21]. Thus, strategies have been proposed to combat turbulence [22,23] and detect divergent vortices [24]. Furthermore, the transmission capacity of the SDM system is mainly determined by the number of available spatial positions and spatially orthogonal modes [25]. As the implementation of optical interconnects is fundamentally limited by available space [26], the data transmission capability of SDM depends on the number of spatially orthogonal modes that can be used in the communication system. Although the OAM modes are infinite in theory, the available OAM modes are limited by actual situations, including the generation, propagation, and detection of OAM beams with large TCs [27,28,29]. Therefore, researchers have tried to increase the transmission capacity of free space optical communication with a small range of OAM modes. For example, Xie and coworkers proposed employing Laguerre–Gaussian (LG) beams with different radial indices to extend the applicable spatial modes [30]. Furthermore, using a fractional vortex in a certain TC range could also increase the number of available states [31].

In addition to LG beams, Bessel beams can also be used in optical communications [32]. In particular, Bessel beams have the characteristic of a diffraction-free optical field with a sharply defined central maximum, which gives them the ability to recover by themselves in the face of obstructions [33]. This makes the Bessel beam have unique advantages when applied to free-space optical communication systems [34]. Perfect vortex (PV), which has an annular ring with a radius and thickness independent of the TC, is well known as the Fourier transform of the Bessel beam [35]. Although PVs are mostly used in optical fiber communications to improve the coupling efficiency when launching OAM beams into fibers [36], they can be collimated and transmitted in free space with the assistance of a microscope objective and a simple lens [37]. Since then, researchers have studied its transmission characteristics and developed several wireless communication links both in free-space and underwater turbulent environments [38,39,40,41,42,43,44]. Furthermore, fractional PVs with half-integer TC were created to multiply the available states of the PVs [45]. Double-ring perfect vortex was generated to increase the number of multiplexing channels [46]. However, these schemes have limited improvement in the available spatial positions and the number of available OAM states. Furthermore, they did not consider the demodulation of each PV ring and the impact of atmospheric turbulence on the channels.

In this work, we proposed a dense space-division multiplexing (DSDM) scheme to increase the transmission capacity and transmission capacity density of free space optical communications with a small range of OAM modes exploiting a multi-ring perfect vortex (MRPV). The pixel checkerboard complex amplitude modulation method (PCAM) that simultaneously modulates the amplitude and phase is employed to engineer an MRPV with a nonoverlapping multi-ring-shaped intensity profile, which consists of four concentric PVs with independently controllable radii, TCs, and amplitudes. The four rings of MRPV are independent spatial channels, which can transport OAM with almost no influence on each other. At the receiver, the four rings of the MRPV can be separated with a multilayer annular aperture, and the OAM mode of each ring can be demodulated. In addition, to demonstrate the transmission stability of the channels of the MRPV, we investigated its performance under different turbulence conditions. The results showed that MRPVs can transport stably under weak turbulence, and the OAM transported by the four circular channels can be separated with tolerable crosstalk. Under the condition of limited available space and OAM states, the DSDM strategy exploiting MRPV might inspire wide optical communication applications exploiting the space dimension of light beams.

## 2. Concept and Principle

**Figure 1:**
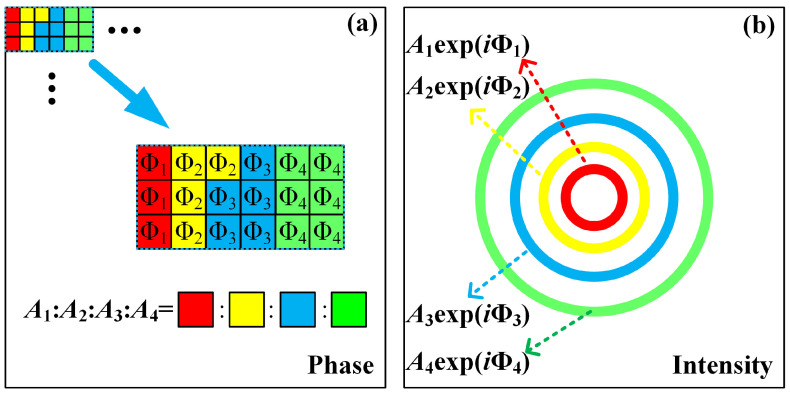
The schematic diagrams of (**a**) the pixel checkerboard complex amplitude modulation method and (**b**) the optical field with controllable amplitude and phase.

To engineer the MRPV, we present a method to encode the amplitude and phase of an optical field into a phase-only hologram. The method is named the PCAM, and its principle is shown in Figure 1. As shown in Figure 1a, the phase-only hologram is divided into many single pixel-sized checkerboards. Several checkerboards form a group, and the hologram is uniformly filled with the group. The red, yellow, blue, and green checkerboards in each group are filled with phases Φ_1_, Φ_2_, Φ_3_, and Φ_4_, respectively. By using this hologram, four beams with additional phases Φ_1_, Φ_2_, Φ_3_, and Φ_4_ could be generated. The number of concolorous checkerboards is proportional to the amplitude of the corresponding beam. Thus, we can ultimately obtain the MRPV, as shown in Figure 1b, with an optical field that can be simplified as follows:

(1) $$E=A_{1}\exp(i\Phi_{1})+A_{2}\exp(i\Phi_{2})+A_{3}\exp(i\Phi_{3})+A_{4}\exp(i\Phi_{4}),$$

where *A*_1_, *A*_2_, *A*_3_, *A*_4_, and Φ_1_, Φ_2_, Φ_3_, Φ_4_ are the amplitudes and phases of the four beams. By changing the number of concolorous checkerboards, we can independently control the amplitude of the corresponding beam.

Now let us consider the phase Φ in each checkerboard. The PV can be obtained via an optical Fourier transform of the Bessel beam [35]. The electric field of an ideal Bessel beam can be expressed as follows:

(2) $$E(r,\phi ,z)=J_{l}(k_{r}r)\exp(il\phi +ik_{z}z),$$

where *J_l_* is the *l*-th Bessel function, and *l* is the TC of the Bessel beam. *k_r_* and *k_z_* are the radial and longitudinal wavenumbers, respectively. *r*, *ϕ*, and *z* are the normalized cylindrical coordinates. The PV is generated at the focus of the Fourier lens, with a field that could be expressed as follows:

(3) $$E(r,\phi )=\frac{i^{l-1}}{k_{r}}\delta (r-R)\exp(il\phi ).$$

where *l* and *R* = *k_r_f*/*k* are the TC and radius of the PV, respectively. *f* is the focal length of the Fourier lens. *k* is the light’s wavenumber. Therefore, the TC and radius of the PV could be manipulated by changing the TC and *k_r_* of the Bessel beam.

Under laboratory conditions, the Bessel beam is produced via the phase mask that combines an axicon phase and a spiral phase:

(4) $$\exp(i\Phi )=\exp\left[i\left(\eta r+l\phi \right)\right],$$

where *η* is the axicon parameter. The radial and longitudinal wavenumbers of the Bessel beam are controlled with the following relationship:

(5) $$\eta =k\tan^{-1}(k_{r}/k_{z})=k\sin^{-1}(k_{r}/k)=k\cos^{-1}(k_{z}/k).$$

Therefore, a PV with controllable TC and radius is obtained by adjusting the values of *l* and *η*. By loading phases Φ_1_, Φ_2_, Φ_3_, and Φ_4_ calculated using Eq. (4) into the four colored checkerboards of the phase-only hologram, four PVs with independently controllable TCs and radii can be obtained.

**Figure 2:**
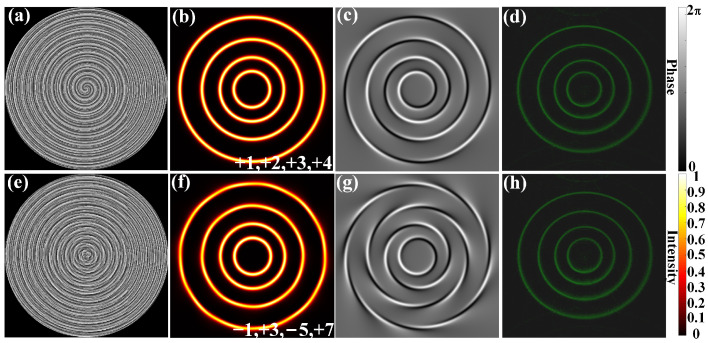
The MRPV with TCs *l*_1_ = 1, *l*_2_ = 2, *l*_3_ = 3, and *l*_4_ = 4 and its (**a**) phase mask, (**b**) transverse intensity profile, (**c**) interference pattern with a spherical wave, (**d**) experimental pattern. The MRPV with *l*_1_ = −1, *l*_2_ = 3, *l*_3_ = −5, and *l*_4_ = 7, and its (**e**) phase mask, (**f**) transverse intensity profile, (**g**) interference pattern with a spherical wave, (**h**) experimental pattern.

Based on the PCAM, the proposed MRPV could be obtained. The TCs of the four rings of MRPV are marked as *l*_1_, *l*_2_, *l*_3_, and *l*_4_ from inside out. As shown in Figure 2a, a phase mask is used to generate an MRPV with TCs *l*_1_ = 1, *l*_2_ = 2, *l*_3_ = 3, and *l*_4_ = 4. The amplitude ratio of the four PVs in the MRPV is set to 3:4:5:6. This means that each group of checkerboards in the phase mask contains 3 red checkerboards, 4 yellow checkerboards, 5 blue checkerboards, and 6 green checkerboards. Furthermore, the four rings of the MRPV should be adjusted to have a uniform power distribution, as the communication system performance could be improved by improving the uniformity of the power distribution of the light field [47]. As the whole intensity of each PV should be the square of the corresponding amplitude, the intensity ratio of the four PVs is 9:16:25:36. Because the intensity of the PV is concentrated in the vicinity of its radius, when the radii of the four PVs are proportional to 9:16:25:36, the four rings of the MRPV will have a uniform power distribution on the transverse intensity profile. The simulation result of the MRPV is shown in Figure 2b. We see that all four rings of MRPV have a nice circular shape and bright intensity of the same magnitude, which conforms to the design objective. Figure 2c shows the interference pattern with a spherical wave, which can be used to verify the OAM mode of each ring in the MRPV by calculating the number of interference fringes. Furthermore, it is also proven by the interference pattern that the OAM modes carried by each ring do not affect each other. Figure 2d shows the MRPV captured in the laboratory using the phase mask in Figure 2a. By comparing the simulation results with the experimental results, the effect of the phase mask generated via the PCAM is verified. The phase mask shown in Figure 2e could be used to generate an MRPV with TCs *l*_1_ = −1, *l*_2_ = 3, *l*_3_ = −5, and *l*_4_ = 7. The simulation results of the MRPV generated via the phase mask and the interference pattern with a spherical wave are shown in Figure 2f,g. Through the number of interference fringes, we can see the magnitude of the TCs. Furthermore, the interference fringes in the interferograms change from clockwise rotation to counterclockwise rotation when the TC is negative. This further confirms that our method can generate the correct PV instead of its conjugate mode. Figure 2h shows the intensity pattern of MRPV obtained in the experiment, which meets well with our expectations. Compared to the method of controlling the amplitude of two beams by randomly distributing pixel positions [48], PCAM is more suitable for generating arrays with more focus.

## 3. Result and Discussion

**Figure 3:**
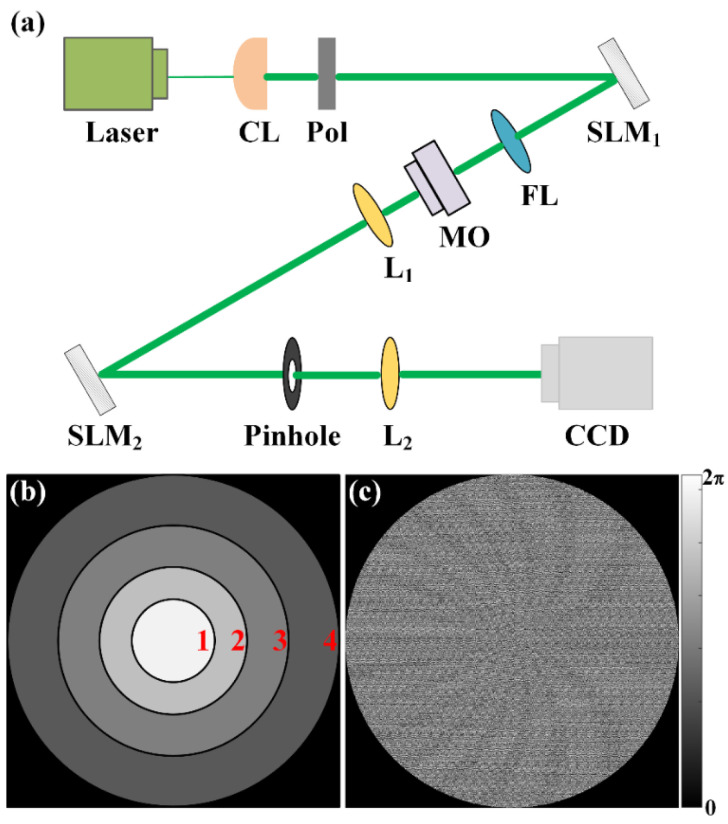
(**a**) Schematic diagram of the experimental setup, (**b**) multilayer annular aperture, (**c**) the phase of the demultiplexing array.

In the previous section, we introduced the method of generating MRPV. The MRPV provides four spatial channels to transmit OAM beams independently at one transverse plane, which is an ideal dense space division multiplexing scheme. Since perfect vortices can transmit stably in free space [37,41], the demodulation of the four channels of MRPV at the receiver becomes the key to the scheme. To this end, we built the experimental system as shown in Figure 3a. A linearly polarized laser beam generated via a diode-pumped solid-state laser (MGL-S-532, 532 nm) is expanded and collimated with a collimator (CL). Then, the beam is modulated with a polarizer to filter the polarization. A phase-only spatial light modulator (SLM_1_, UPOLabs, HDSLM80R-PLUS, 1920 × 1200 pixels, pixel pitch 8 μm, 60 Hz) is illuminated by the incident beam. The phase mask uploaded on SLM_1_ is used to generate the multiplexing Bessel beams. Then, a Fourier lens (FL) is employed to transform the Bessel beams into an MRPV. The microscope objective (MO) placed at the focal point of FL and the lens (L_1_) placed behind the MO is employed to collimate the MRPV. The demultiplexing of the MRPV is performed by SLM_2_, which is achieved via the collaboration of two phases. A multilayer annular aperture is employed to filter the four rings of the MRPV. As shown in Figure 3b, the boundary of the multilayer annular aperture is the middle of the two adjacent PV rings. Therefore, the multilayer annular aperture is divided into four regions. In each region, the transmittance function of the aperture is filled with a specially designed blazed grating. By changing the transverse and longitudinal period coefficients of the two-dimensional blazed grating, it is possible to shift the four OAM rings of the MRPV to the positive direction of the *x*-axis, the negative direction of the *x*-axis, the positive direction of the *y*-axis, and the negative direction of the *y*-axis, respectively. When detecting the mode of MRPV, the four rings of MRPV are extracted separately with the multilayer annular aperture. Then, a vortex demultiplexing array (its phase is shown in Figure 3c) is employed to demultiplex the OAM mode of a single PV ring. There are 15 spots with inherent OAM states from −7 to +7 in the array. When detecting the OAM beam with a TC that is *l* transmitted via the spatial channel, the vortex spot with inherent TC −*l* will be transformed into a Gaussian-like spot. Finally, the demultiplexing array is collected using a charge-coupled device (CCD, Basler, acA4112, 4096 × 3000 pixels, pixel size of 3.45 μm, 30 Hz), which is placed at the focal plane of lens L_2_. The pinhole behind lens L_2_ is used to select the corresponding diffraction order.

**Figure 4:**
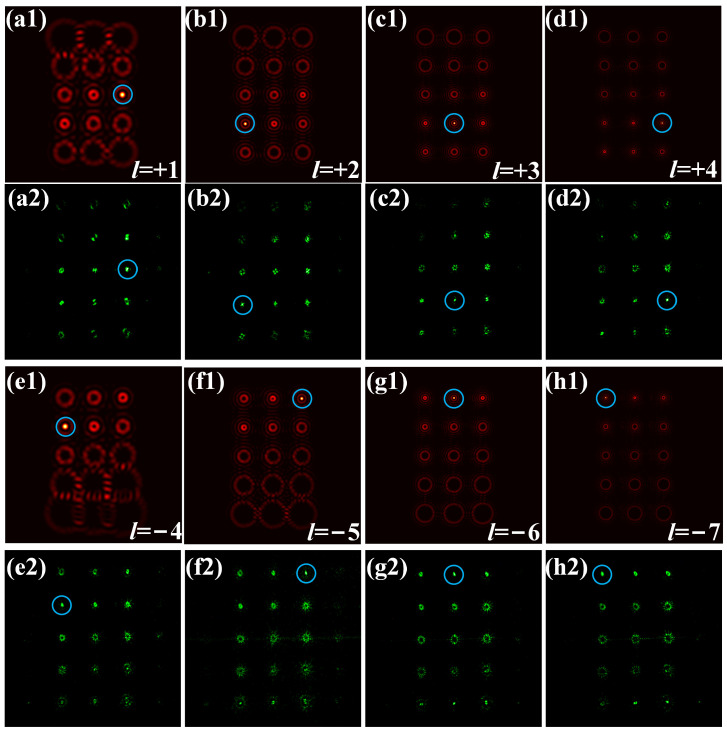
Demultiplexing of MRPVs through simulation and experiment. (**a1**–**d1**), (**a2**–**d2**) the MRPV with TCs *l*_1_ = 1, *l*_2_ = 2, *l*_3_ = 3, and *l*_4_ = 4, (**e1**–**h1**), (**e2**–**h2**) the MRPV with TCs *l*_1_ = −4, *l*_2_ = −5, *l*_3_ = −6, and *l*_4_ = −7. The incident OAM states are marked by the bule circles.

Two MRPVs with TCs *l*_1_ = 1, *l*_2_ = 2, *l*_3_ = 3, and *l*_4_ = 4 and TCs *l*_1_ = −4, *l*_2_ = −5, *l*_3_ = −6, and *l*_4_ = −7 are demultiplexed as examples. The simulation and experimental results are shown in Figure 4. In each row, the four pictures from left to right show the OAM demultiplexing results of the four spatial channels carried by the MRPV. The demultiplexing result of the MRPV with TCs *l*_1_ = 1, *l*_2_ = 2, *l*_3_ = 3, and *l*_4_ = 4 is shown in Figure 4(a1–d1). According to the position of the spot transformed to a Gaussian-like spot in the demultiplexing array, the mode of the OAM beam transmitted in the four rings of the MRPV can be deduced directly as *l*_1_ = 1, *l*_2_ = 2, *l*_3_ = 3, and *l*_4_ = 4. The OAM channels carried by the MRPV are also measured experimentally. As shown in Figure 4(a2–d2), the experimental results are consistent with the simulation. The simulation and experimental results of the demultiplexing of the other MRPV are shown in Figure 4(a3–d3) and Figure 4(a4–d4), respectively. We can see that the spots in the demultiplexing arrays with original TCs of 4, 5, 6, and 7 are transformed into Gaussian-like spots. Thus, the modes of OAM beams transmitted by the four rings are *l*_1_ = −4, *l*_2_ = −5, *l*_3_ = −6, and *l*_4_ = −7. From the experimental results, it can be seen that the other modes are not detected in the demultiplexing arrays, indicating that the impact between the four rings of MRPV is very small, which also proves the reliability of the method for generating MRPV. In general, the demultiplexing of MRPVs can be achieved using a multilayer annular aperture and a vortex demultiplexing array.

In addition, we analyzed the separation of MRPV channels after atmospheric turbulence. When a beam propagates through the atmosphere, the variations in the refractive index along the optical path caused by the temperature and pressure inhomogeneities eventually lead to random distortion of the beam’s wavefront. Therefore, the influence of atmospheric turbulence should be considered in the demultiplexing of MRPVs. Here, the turbulence model developed by von Karman is employed, in which the power spectral density of the refractive index fluctuation is described in [20,49].

(6) $$\phi_{n}(\kappa )=0.033C_{n}^{2}{\left(\kappa^{2}+k_{0}^{2}\right)}^{-11/6}\exp(-\kappa^{2}/k_{m}^{2}).$$

where *k*_0_ = 2π/*L*_0_ and *k_m_* = 5.92/*l*_0_. Parameters *L*_0_ and *l*_0_ are the outer and inner scales of turbulence, respectively. $C_{n}^{2}$ is the refractive index structure parameter. *κ* is the angular spatial frequency. The phase spectrum could be calculated using the power spectral density of the refractive index fluctuation as follows:

(7) $$\phi_{\varphi }(\kappa )=2\pi k^{2}z\phi_{n}(\kappa ).$$

where *k* is the wavenumber of the incident beam. *z* is the propagation distance in the turbulence. Then, the phase distribution of atmospheric turbulence can be obtained as follows:

(8) $$\varphi =F\left[C\left(\frac{2\pi }{N\Delta x}\right)\sqrt{\phi_{\varphi }\left(\kappa \right)}\right].$$

where *C* is an *N* × *N* array with complex random numbers with zero mean and variance 1. Δ*x* is the grid spacing and *F* denotes the Fourier transformation.

**Figure 5:**
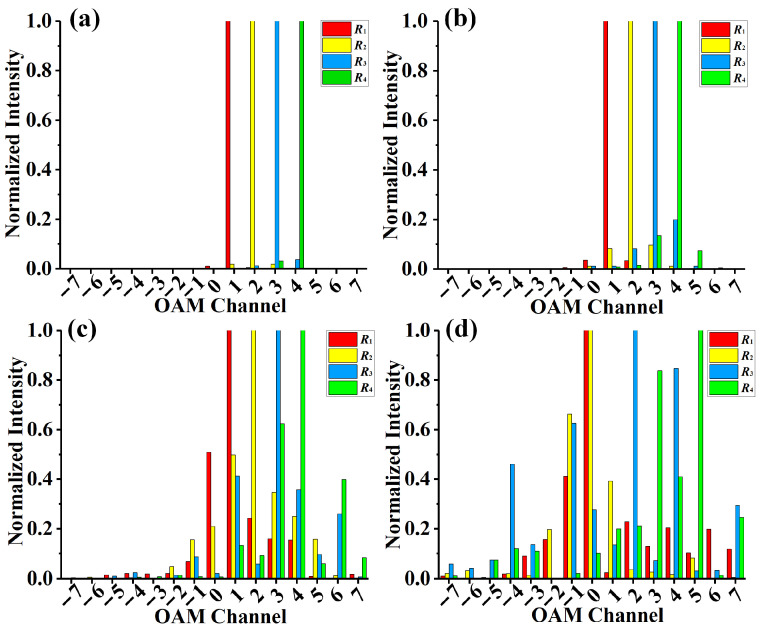
Demultiplexing of the MRPV (**a**) before transmission, (**b**) with weak turbulence, (**c**) with moderate turbulence, (**d**) with strong turbulence.

Using this method, three phase-only screens representing atmospheric turbulence with different turbulence strengths are constructed. When the phase screen is loaded into SLM, the transmission of MRPV in atmospheric turbulence can be simulated. The demultiplexing of the MRPV before and after propagating through the atmospheric turbulence at a distance of 100 m with different refractive index structure parameters $C_{n}^{2}$ is shown in Figure 5, in which $C_{n}^{2}$ is 1 × 10^−13^ m^−2/3^ for strong turbulence, 1 × 10^−14^ m^−2/3^ for moderate turbulence, and 1 × 10^−15^ m^−2/3^ for weak turbulence. The OAM modes of the four rings carried by the MRPV are *l*_1_ = 1, *l*_2_ = 2, *l*_3_ = 3, and *l*_4_ = 4. In the histogram, the red, yellow, blue, and green areas represent the OAM beams measured in the first, second, third, and fourth rings, respectively. From Figure 5a, we can see that when the MRPV is directly measured without propagating in atmospheric turbulence, OAM beams transmitted in the four rings carried by MRPV can be demodulated with faint inter-ring crosstalk. However, when the MRPV propagates in turbulence, its wavefront is distorted, which causes the demodulation error of the MRPV. As shown in Figure 5b–d, with increasing turbulence strength, the crosstalk that arises in the demultiplexing of the MRPV increases. Under weak turbulence, the influence of turbulence on the four rings carried by the MRPV is small, and the OAM beams transmitted via the four channels can be separated effectively. Furthermore, as shown in Figure 5b, we can see that the mode crosstalk of the inner vortex ring is relatively smaller than that of the outer vortex ring. This indicates that a vortex ring with a larger size will suffer from more severe phase distortion. It is particularly noteworthy that turbulence also has a stronger impact on beams with higher vortex orders [50]. When the MRPV propagates in moderate turbulence, the OAM beams transmitted in four spatial channels will exhibit obvious wavefront distortion, which leads to large crosstalk in the demodulation of the MRPV. At this time, the outer ring is still more severely affected than the inner ring, as its energy not only leaks largely into the adjacent first-order OAM modes but also leaks largely into the adjacent second-order OAM modes. Even so, the OAM beams transmitted via the four spatial channels can still be demodulated. However, when the MRPV propagates in strong turbulence, it is difficult to identify the OAM beam transmitted via the spatial channels carried by the MRPV because substantial crosstalk appears. In general, the four channels of MRPV could be measured and demodulated directly under appropriate turbulence. When encountering stronger turbulence, it is necessary to use appropriate methods to combat turbulence.

## 4. Conclusions

In this work, we proposed a DSDM scheme exploiting the MRPV, which consists of four PVs with different radii. The MRPV provides four spatial channels to transmit OAM beams under the condition of occupying only one space position. By using the PCAM, which adjusts the amplitude and phase of the light field simultaneously using a phase-only hologram, the amplitude, TC, and radius of each ring in the MRPV can be freely modulated. At the receiver, the four spatial channels carried by the MRPV can be separated using a multilayer annular aperture. The experimental results show that the inter-ring crosstalk between the four channels of MRPV is very small, and the OAM mode transmitted in these spatial channels can be efficiently demodulated. In addition, the stability of the four MRPV channels can be preserved under appropriate turbulence conditions. We believe that the proposed MRPV can be applied to increase the transmission capacity of free-space communication systems with few available OAM modes. The proposed PCAM can not only modulate perfect vortices but also be applied to the multiplexing of other beams such as Laguerre–Gaussian beams.

## Data Availability

The simulated and experimental data that support the works of this study are available from the corresponding authors upon reasonable request.
